# Association between cognitive impairment and poor antihypertensive medication adherence in elderly hypertensive patients without dementia

**DOI:** 10.1038/s41598-018-29974-7

**Published:** 2018-08-03

**Authors:** Mi Hee Cho, Dong Wook Shin, Sung-A Chang, Ji Eun Lee, Su-Min Jeong, Sang Hyuck Kim, Jae Moon Yun, Kiyoung Son

**Affiliations:** 1 Department of Family Medicine, Seoul National University Health Service Center, 1, Gwanak-ro, Gwanak-gu, Seoul 08826 Republic of Korea; 2 Department of Family Medicine/Supportive Care Center, Samsung Medical Center, 81, Irwon-Ro, Gangnam-gu, Seoul 06351 Republic of Korea; 3 Division of Cardiology, Department of Medicine, Heart Vascular Stroke Institute, Samsung Medical Center, Sungkyunkwan University School of Medicine, 81, Irwon-Ro, Gangnam-gu, Seoul 06351 Republic of Korea; 4 Department of Family Medicine, Seoul National University Hospital, 101, Daehak-ro, Jongno-gu, Seoul 03080 Republic of Korea

## Abstract

Adherence to antihypertensive medication is a critical factor to control blood pressure and prevent complications. However, cognitive impairment can negatively affect medication adherence. In this study, we investigated the association between cognitive function and antihypertensive medication adherence among elderly hypertensive patients using the Korean National Health Insurance Service National Sample Cohort Data of the Elderly Cohort. The study included 20,071 elderly hypertensive patients and the prevalence of poor medication adherence to antihypertensive medications was 16.4%. A multivariate logistic regression analysis showed that lower cognitive function was associated with poor medication adherence (adjusted odds ratio 0.980, 95% confidence interval 0.961–0.999). Additionally, high income levels, living in metropolitan areas, and comorbidities (such as stroke, coronary heart disease, diabetes, and dyslipidemia) were positively associated with medication adherence, while patients diagnosed with cancers showed poor medication adherence. Our study demonstrated that cognitive impairment is a possible risk factor for poor antihypertensive medication adherence, even in patients without dementia. Thus, clinicians involved with geriatric care should monitor patients’ cognitive function and medication adherence. And if a patient shows cognitive impairment, clinicians need to educate patients and caregivers about the importance of proper adherence, and consider proper interventions to optimize the cognitive function of elderly patients.

## Introduction

Patients with hypertension need life-long treatment with antihypertensive medications to prevent cardiovascular diseases (CVD)^1,2^. Proper adherence to antihypertensive medications improves a patient’s chance of maintaining a target blood pressure^3,4^. However, poor medication adherence is commonly observed in hypertensive patients. It is associated with high CVD incidence and mortality^5^, hospitalization, and high health care expenditures^6–8^. Elderly patients with chronic diseases are particularly likely to show poor medication adherence compared to younger patients^2,9^. Several factors, including polypharmacy, a poor doctor-patient relationship, and cognitive impairment have been mentioned as barriers to medication adherence in elderly patients^9^.

Cognitive impairment can negatively affect medication adherence in elderly patients. The daily and regular intake of antihypertensive medication requires a certain level of cognitive function, such as a good attention span and working memory^10^. It is well known that people with dementia show poor adherence to medication^11,12^, including antihypertensive medication^12^. However, for the non-demented elderly, reported findings have been inconclusive^9,10,13–18^. While some studies have reported a strong association between cognitive function and medication adherence^14–16^, others have not demonstrated a significant relationship between them^17^. Additionally, measurements of medication adherence in these studies were mostly based on monitoring pill counts in patients^14–16^, or via self-reports obtained from patients or caregivers^17,18^. This could have produced biased results due to patient awareness of being monitored.

Thus, we designed a retrospective cohort study using a national administrative database in order to investigate the association between cognitive function and antihypertensive medication adherence among elderly hypertensive patients without dementia in Korea. We hypothesized that the use of routinely collected prescription data would minimize the bias linked to the direct monitoring of adherence, and would yield more representative results than previous studies.

## Results

### Study participants

**Figure 1:**
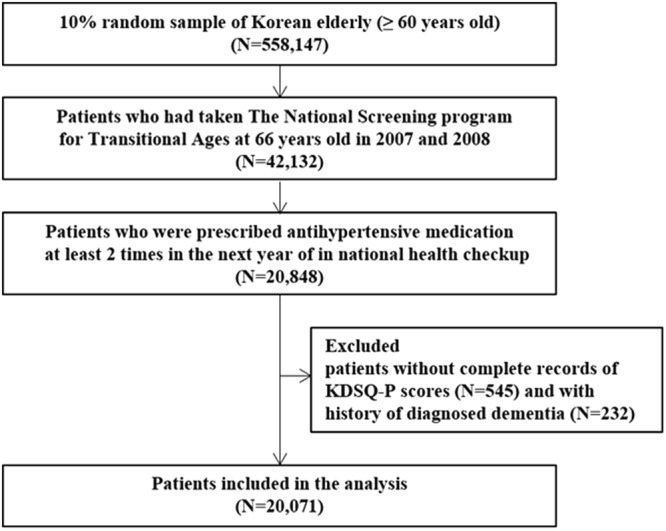
Study population. KDSQ-P, Prescreening Korean Dementia Screening Questionnaire.

We found that 20,848 elderly patients had been prescribed antihypertensive medication at least twice in the next year of the National Screening Program for Transitional Ages (NSPTA). After excluding patients without complete records regarding cognitive function and those with a history of diagnosed dementia, 20,071 hypertensive patients were included in the study population (Fig. 1). Among these elderly hypertensive patients, the prevalence of poor adherence to antihypertensive medications was 16.4%. Many basic demographic and clinical characteristics were significantly different between the poor and good adherence group, with the exception of sex (Table 1). Using a univariate analysis, we found that results obtained from screening tests for depression and cognitive function were not statistically different between the two groups (Table 1).

**Table 1:** Demographic and clinical characteristics of elderly patients with hypertension

	Antihypertensive Medication Adherence						
	Total		Poor adherence		Good adherence		*p*
	N	%*	N	%**	N	%**	
Total N for analysis	20,071	100	3,297	16.4	16,774	83.6	
**Sex**							
Male	9,165	45.7	1,465	16.0	7,700	84.0	0.121
Female	10,906	54.3	1,832	16.8	9,074	83.2	
**Residential area**							
Metropolitan	4,459	22.2	574	12.9	3,885	87.1	<0.001
City	5,037	25.1	892	17.7	4,145	82.3	
Rural	10,575	52.7	1,831	17.3	8,744	82.7	
**Income grade**							
Grade 0~4 (low)	6,198	30.9	1,065	17.2	5,133	82.8	0.007
Grade 5~8 (middle)	7,634	38.0	1,282	16.8	6,352	31.2	
Grade 9~10 (high)	6,239	31.1	950	15.2	5,289	84.8	
**Comorbidity**							
Stroke	1,446	7.2	188	13.0	1,258	87.0	<0.001
Coronary heart disease	2,864	14.3	342	11.9	2,532	88.1	<0.001
Diabetes	5,334	26.6	634	11.9	4,799	88.1	<0.001
Dyslipidemia	4,598	22.9	550	12.0	4,048	88.0	<0.001
Cancer	1,108	5.5	209	18.9	899	81.1	0.024
	**Mean**	**SD**	**Mean**	**SD**	**Mean**	**SD**	***p***
GDS score	0.75	0.90	0.76	0.88	0.75	0.90	0.728
KDSQ-P score	1.77	1.97	1.81	2.00	1.76	1.96	0.170

SD, standard deviation; GDS, Geriatric Depression Screening (range 0–3); KDSQ–P, Prescreening Korean Dementia Screening Questionnaire (range 0–10).

*Column percentage; **Row percentage.

### Factors associated with poor adherence

A multivariate logistic regression analysis demonstrated that low cognitive function was associated with poor medication adherence, and the adjusted odds ratio [aOR] was 0.980 per score of the Prescreening Korean Dementia Screening Questionnaire (KDSQ-P) (95% confidence interval [CI] 0.961–0.999) (Table 2). Additionally, antihypertensive medication adherence in elderly patients living in small cities (aOR 0.706, 95% CI 0.630–0.792) and rural areas (aOR 0.729, 95% CI 0.658–0.807) was lower than that demonstrated by patients living in metropolitan areas (Table 2). Patients with comorbidities including stroke, coronary heart disease, diabetes, and dyslipidemia showed a positive association with good medication adherence, while patients diagnosed with cancers as a comorbidity were shown to have a poor medication adherence (aOR 0.802, 95% CI 0.686–0.939) (Table 2). A high score on the Geriatric Depression Scale (GDS) showed no significant association with medication adherence (aOR 0.988, 95% CI 0.947–1.031) (Table 2).

**Table 2:** Multivariate logistic regression analysis for association between cognitive function and medication adherence.

	All subjects (N = 20,071)		
	Odds ratio	95% CI	*p*
**Sex**			
Male	1.000	—	—
Female	0.929	0.860–1.004	0.063
**Residential area**			
Metropolitan	1.000	—	—
City	0.706	0.630–0.792	<0.001
Rural	0.729	0.658–0.807	<0.001
**Income grade**			
Grade 0~4 (low)	1.000	—	—
Grade 5~8 (middle)	1.021	0.933–1.117	0.651
Grade 9~10 (high)	1.126	1.022–1.241	0.016
**Comorbidity**			
Stroke	1.351	1.151–1.585	<0.001
CHD	1.501	1.330–1.694	<0.001
Diabetes	1.639	1.492–1.800	<0.001
Dyslipidemia	1.533	1.388–1.693	<0.001
Cancer	0.802	0.686–0.939	0.006
GDS Score	0.988	0.947–1.031	0.584
KDSQ-P Score	0.980	0.961–0.999	0.042

CHD, coronary heart disease; GDS, Geriatric Depression Scale (range 0–3); KDSQ-P, Prescreening Korean Dementia Screening Questionnaire (range 0–10).

## Discussion

As society ages, healthcare expenditures dramatically rise (not only in Korea, but also in several developed countries). South Korea is predicted to become a super-aging society by 2025, with over 20% of its population ≥65 years of age. Because poor medication adherence can lead to unexpected hospitalization and subsequent costs, improvement of medication adherence in elderly patients is important.

To the best of our knowledge, this study is the first to investigate the association between mild cognitive impairment and medication adherence using routinely collected prescription data for elderly hypertensive patients without overt dementia. The greatest advantage to using prescription data gathered from national administrative registries is that the data more accurately reflect the real-world scenario pertaining to medication adherence. This is because there is no monitoring involved (*e.g*. self-reporting or pill counts), which can significantly affect a patient’s behavior. Other strengths of our study include a large-sized, nationwide study population and a rigorous adjustment for various comorbid diseases.

The present study demonstrates that medication adherence worsens with a decline in cognitive function, even in patients without overt dementia. The adjusted odds ratio of poor medication adherence was 0.980 per score of the screening cognitive test. We can transfer our results into the clinical context. For example, if a patient scores five points on the KSDQ scale (which is just above the cut-off point), he or she is likely to have 10% lower adherence than a patient with intact cognition (*e.g*. the KSDQ score of 0). Poor medication adherence associated with mild cognitive impairment can be attributed to several factors. Damaged cognitive function results in unintentional medication nonadherence^19,20^. Memory is one of the most essential cognitive functions needed to plan, organize, and execute medication administration with respect to correct timing and dosage, based on a patient’s prescription^15^. A decline in executive function and working memory in patients with cognitive impairment is an important risk factor associated with nonadherence^21^. Further studies will be required to explore the mechanism why patients with mild cognitive impairment have difficulties adhering to medication.

The association between medication adherence and cognitive function demonstrated in this study carries important clinical implications. First, clinicians involved with geriatric care should thoroughly evaluate cognitive function in elderly patients, and spend time and effort in educating patients with mild cognitive impairment and their caregivers about the significant risks associated with inappropriate medication adherence. A systematic review has reported that education regarding the potential risk of disease can improve medication adherence^9^. Second, our study suggests that a focus on improved cognitive function may be a useful strategy to improve medication adherence. Cognitive function is a potentially modifiable factor. Previous studies have reported that cognitive function can be preserved or improved through various interventions, such as cognitive training or the administration of cholinesterase inhibitors^22–24^. Behavioral therapy, such as cognitive training, showed efficacy in a recent systematic review (although with varying degrees of effectiveness^22,23^). Although the benefits of pharmacological intervention are debatable^22–25^, studies have demonstrated that medications such as cholinesterase inhibitors can delay progression to Alzheimer’s disease^26^. Thus, there is the potential that such cognitive interventions can improve medication adherence in patients with mild cognitive impairment (MCI), although further studies are needed. Furthermore, the use of advanced technology to improve medication adherence (*e.g*. smartphone applications^27^, smart pill boxes^28,29^, or drugs with ingestible drug sensors^30^) could be tried for those who show mild cognitive impairment and frequently forget to take medication.

Our study highlighted other factors related to medication adherence. A high income and residing in a metropolitan area were factors that were positively associated with antihypertensive medication adherence, consistent with findings reported previously^31^. Easy access to medical institutions/health care facilities in metropolitan areas and lower drug cost burdens for high-income patients may each contribute to adherence. Patients with other comorbid non-cancerous conditions also showed increased medication adherence, consistent with results observed in previous studies^31^. While having comorbidities could be related to polypharmacy problems (which have been linked with poor adherence), our data suggest that patients with non-cancerous comorbidity are more alert to their risk of developing serious disease. In contrast, medication adherence in elderly patients with cancers was low. This finding is consistent with previous reports suggesting relative indifference toward non-cancer health needs in these patients^31^.

Notwithstanding its strengths and usefulness, the limitations of our study are as follows: 1) The database for this study included only patients who voluntarily attended the national health screening program, leading to selection bias, also called as ‘screenee effect’. Participants involved in health screening programs are generally more attentive to their health compared to the general population. This fact might explain the higher rates of adherence to antihypertensive agents observed in this study compared to previous studies^6,31^. Moreover, patients with very severe degrees of cognitive impairment are unlikely to participate in a screening program, and are potentially underrepresented in our study. 2) We estimated medication adherence using cumulative medication adherence (CMA) methods based on administrative data. Thus, we could not verify whether patients actually bought and used medications, which could have resulted in an underestimation of poor medication adherence. However, currently, there is no absolute measure or standard to estimate medication adherence, and CMA methods are considered less biased than self-reports^32^. 3) Due to the nature of the administrative database, our dataset lacked clinical details. Reasons for poor medication adherence (such as side effects of medication, polypharmacy, or living without caregivers) remain unknown. 4) Although the KDSQ was relatively not impacted by education level, the education background of each patient should have been considered in order to evaluate cognitive function. However, this information was not available in the database used in this study. 5) The measure of cognitive function we used is helpful for quick mass-screening, and is simpler to use than other established measures such as the Mini-Mental State Examination (MMSE)^33^ or the Montreal Cognitive Assessment (MoCA)^34^. Nevertheless, our mass-screening method has demonstrable efficacy and has been validated to identify elderly people who need further examination for dementia^35^. 6) Our study was performed in an Asian country. Therefore, extrapolation of our results to other populations requires caution. In Asia, elderly adults usually live with their children and/or family. As caregivers, family members look after needs during illness and help patients comply with their medications^36^. This might not be the case in Western countries, where elderly adults often maintain an independent lifestyle. In the latter case, cognitive dysfunction is likely to manifest with a more profound effect.

In conclusion, we found that mild cognitive impairment is a possible risk factor associated with poor antihypertensive medication adherence, even in patients without dementia. Thus, clinicians involved with geriatric care should monitor patients’ cognitive function and medication adherence. If a patient shows cognitive impairment, clinicians need to educate both the patient and caregiver about the importance of proper adherence. Clinicians should also consider interventions that optimize the cognitive function of elderly patients.

## Methods

### Study setting

The National Health Insurance Service (NHIS) provides mandatory universal health insurance to most Koreans (96.9%). As an exception, the lowest-income population is covered by a public-assistance plan (*i.e*. Medicaid). Beneficiaries of the NHIS ≥ 40 years of age are eligible to participate in the National Health Screening Program (NHSP) biennially. The NHSP is primarily designed to identify cardiovascular risk factors, and includes assessment through questionnaires (past medical history, health behavior), an anthropometric exam (height, weight, waist circumference, and blood pressure), and laboratory tests (blood sugar, lipid profile, and creatinine)^37^.

The NSPTA is additionally offered to people at 40 and 66 years of age^38^. For those aged 66 years (in addition to the routine items included in the NHSP), the NSPTA includes a questionnaire to screen for depression (three selected items belonging to GDS^39^), a physical function test (the Timed Up and Go test)^40^, and a brief screening test for cognitive impairment (KDSQ-P)^35^. These tests detect common problems experienced by the elderly, such as depression, frailty, and dementia.

### Data sources

The Korean NHIS database contains medical and demographic information, including results of the National Health Examinations (both the NHSP and NSPTA). A patient’s medical information includes utilization of medical facilities (such as visits to outpatient clinics or hospitalization), registered diagnostic codes (based on International Classification of Disease (ICD) codes), and prescribed medicines (including dates of prescription, amounts dispensed, and the number of defined daily doses^41^). Demographic information includes age, sex, insurance premium (proxy for income level), disability status, and place of residence^41^. This database has been extensively used for epidemiological studies, and its design and usage are described in detail elsewhere^42,43^.

This retrospective cohort study was performed using Korean National Health Insurance Service National Sample Cohort Data of the Elderly Cohort (2002–2013) (NHIS-NCES). The NHIS-NCES database comprises a subset of data found in the NHIS database, and is openly shared for research purposes on request. This database includes approximately 550,000 people in total. They were randomly selected from the total Korean population who were ≥60 years old (about 5.5 million) and covered by the NHIS or the public-assistance plan (Medicaid) on December 31, 2002. Registered patients were followed-up between January 1, 2002 and December 31, 2013.

The Institutional Review Board (IRB) of Seoul National University Hospital approved this study (IRB No. 1611–061–807) and waived the requirement of informed consent from the study participants due to the anonymity of the data obtained from the NHIS database. All experiments were conducted in accordance with the relevant guidelines and regulations.

### Study population

Among participants registered in the NHIS-NCES database, only those who were 66 years old in 2007 or 2008 were included in the NSPTA. Thus, our study population included 42,132 elderly patients (with NSPTA results) out of the 558,147 participants available in the NHIS-NCES database in order to investigate the association between cognitive function and medication adherence. Among these 42,132 patients, we further selected those with a history of being prescribed antihypertensive medications at least twice in the subsequent year of the NSPTA (n = 20,848). Exclusion criteria for our study were as follows: patients without complete records regarding cognitive function tests (n = 545) and those presenting with a history of dementia diagnosed before the date of the health checkup (n = 232).

### Measurements

#### Exposure

Cognitive function was assessed using the KDSQ-P, for which validity is well established^35^. The KDSQ-P was developed by adopting selected questions from the Korean Dementia Screening Questionnaire-Cognition (KDSQ-C). The KDSQ-C shows a significantly inverse correlation with the Korean Mini-Mental State Examination (K-MMSE)^44^. The KDSQ-P is composed of five items, of which four evaluate memory function and the remaining item assesses abilities associated with daily living^35^. Each item is scored 0–2, for a total score range of 0–10^35^. A high score denotes poor cognitive function, and patients with a cut-off score ≥4 undergo further tests for the diagnosis of dementia.

#### Outcomes

Medication adherence to antihypertensive medications was calculated using medication prescription data for one year (the next year of the health exam). We used the cumulative medication adherence (CMA) scale, a standard method of measuring medication adherence using prescription data^45^. Since automatic refills of antihypertensive medication are not allowed in Korea, patients need to visit a clinic to get a prescription from a clinician every time. The CMA scale is defined as the percentage of the number of days for which specific medications are prescribed for a certain period of time. Theoretically, the CMA scale ranges between 0 and 100%^44^. The cut-off value for appropriate adherence was 80% because this has been commonly used as a threshold for medication adherence in patients with hypertension^5,46,47^.

#### Covariates

The NHIS-NCES provides deciles of income level based on National Health Insurance premiums, which were categorized into three income groups (low, middle, and high) in this study. Comorbidities including stroke, coronary heart disease, diabetes, dyslipidemia, and cancers were defined using related ICD-10 codes for each disease. Codes were registered within the previous or same year of the NSPTA. Patients were classified as depressed if they answered ‘Yes’ to any of the three selected items on the GDS (*i.e*. loss of activity or interest, feelings of worthlessness, and feelings of hopelessness).

### Statistical analyses

Characteristics of patients with appropriate medication adherence (CMA ≥ 80%) were compared with those of patients demonstrating poor adherence. Categorical variables were presented as percentages. Continuous variables (such as GDS scores or KDSQ-P scores) were expressed as the mean ± standard deviation (SD). Comparisons between groups were performed using the t-test for continuous variables, and the chi-square test or the Fischer exact test for categorical values, with a univariate analysis. A multivariate logistic regression analysis was performed to analyze the association between cognitive function and medication adherence with adjustment for sex, income level, residential area, comorbidities, and level of depression. All analyses were performed using STATA version 14 for Windows (College Station, TX, USA). A *P*-value < 0.05 was considered statistically significant.

### Data availability

The datasets generated during and/or analyzed during the current study are available from the corresponding author on reasonable request.
